# RNA-seq data from mature male gonads of marine mussels *Mytilus edulis* and *M. galloprovincialis*

**DOI:** 10.1016/j.dib.2018.09.086

**Published:** 2018-10-03

**Authors:** Angel P. Diz, Mónica R. Romero, Andrés Pérez-Figueroa, Willie J. Swanson, David O.F. Skibinski

**Affiliations:** a Department of Biochemistry, Genetics and Immunology, Faculty of Biology, University of Vigo, Vigo, Spain; b Marine Research Centre, University of Vigo (CIM-UVIGO), Isla de Toralla, Vigo, Spain; c Department of Genome Sciences, School of Medicine, University of Washington, Seattle, USA; d Institute of Life Science, Swansea University Medical School, Swansea University, Swansea, UK

**Keywords:** Invertebrates, Mollusks, Mature male gonads, Transcriptomics, Illumina paired-end, *De novo* sequencing

## Abstract

The mussels *Mytilus edulis* and *Mytilus galloprovincialis* are marine organisms with external fertilization able to hybridize where their distributions overlap allowing the study of reproductive isolation mechanisms in nature. We provide raw data of a transcriptomic analysis of mature male gonads from these two *Mytilus* spp. using NGS (Illumina) technology and a preliminary list of transcript that were functionally annotated showing species-specific differential expression. A shortlist including some of these genes and their corresponding proteins have been thoroughly analysed and discussed in Romero et al. (2018, Submitted for publication).

**Specifications table**

**Table:** 

Subject area	*Biology*
More specific subject area	*Evolutionary and Reproductive Biology, Transcriptomics*
Type of data	*Transcriptomics (RNA-seq)*
How data was acquired	*High-throughput sequencing (Illumina HiScanSQ)*
Data format	*Raw (fastq), filtered and analysed*
Experimental factors	*Two closely-related marine mussel species (Mytilus edulis and M. galloprovincialis)*
Experimental features	*6 individual samples from each Mytilus species corresponding to reproductively mature male individuals were chosen for RNA extraction, and total RNA extracts from these samples were used to make two pools of 6 individuals each, one pool for each of the two Mytilus species. mRNA libraries were generated using the Illumina Truseq Small RNA Preparation kit and analysed in two full lines (1 per each pooled sample/species) of the flow cell from an Illumina HiScanSQ instrument.*
Data source location	*Swansea (UK) and Vigo (Spain)*
Data accessibility	*Raw data were deposited into SRA-NCBI database, BioProject ID: PRJNA451093 (*https://www.ncbi.nlm.nih.gov/bioproject/451093*), while results from further analyses are provided as supplementary files in this article.*

**Value of the data**

• Transcriptome data from mature male gonads of two closely related marine mussel species can provide insights into fundamental and functional aspects of reproduction in these species and more broadly in external fertilizers and invertebrates.
• Comparative gene expression data of mature male gonads between *M. edulis* and *M. galloprovincialis* provide a preliminary list of genes with potential involvement in species-specific differences.
• Because these two marine mussel species are able to hybridise where their distribution overlap some of the differential expressed genes could be good targets for further evolutionary studies in relation to the study of reproductive isolation mechanisms that ultimately could lead to speciation (*e.g.* see Romero et al. [1]).
• The present transcriptome database, once is translated to protein sequences, can be further used for tissue and organism-specific proteomic analysis in order to enhance the number and quality of protein identifications through mass spectrometry analysis (*e.g.* see Romero et al. [1]).

## 1 Data

Raw data (100 bp paired-end reads, FASTQ files) resulting from sequencing analysis (Illumina HiScanSQ) of two cDNA libraries each prepared from a pool of equivalent amounts of total RNA extracts from 6 male gonad tissues of *Mytilus edulis* and *Mytilus galloprovincialis* (see details below) were deposited in SRA-NCBI database (BioProject ID: PRJNA451093, BioSample accessions: SAMN08959310, SAMN08959311) (https://www.ncbi.nlm.nih.gov/bioproject/451093). Raw reads were used for *de novo* assembly producing a list of a total of 97,425 isotigs (File S1) that were grouped in 49,713 loci (File S2). File S2 represents the consensus male gonad tissue transcriptome of both *Mytilus* species. Resulting transcripts were functionally annotated using Blast2Go (File S3) and InterProScan (File S4) as described below. A list with the expression levels for each isotig and species and those with differential expression (DE) between the two *Mytilus* species (FDR 5%) according to RSEM analysis are provided in Files S5 and S6 respectively, as well as annotations corresponding to this shortlist of DE isotigs (File S7).

## 2 Experimental design, materials and methods

### 2.1 Samples

**Fig. 1:**
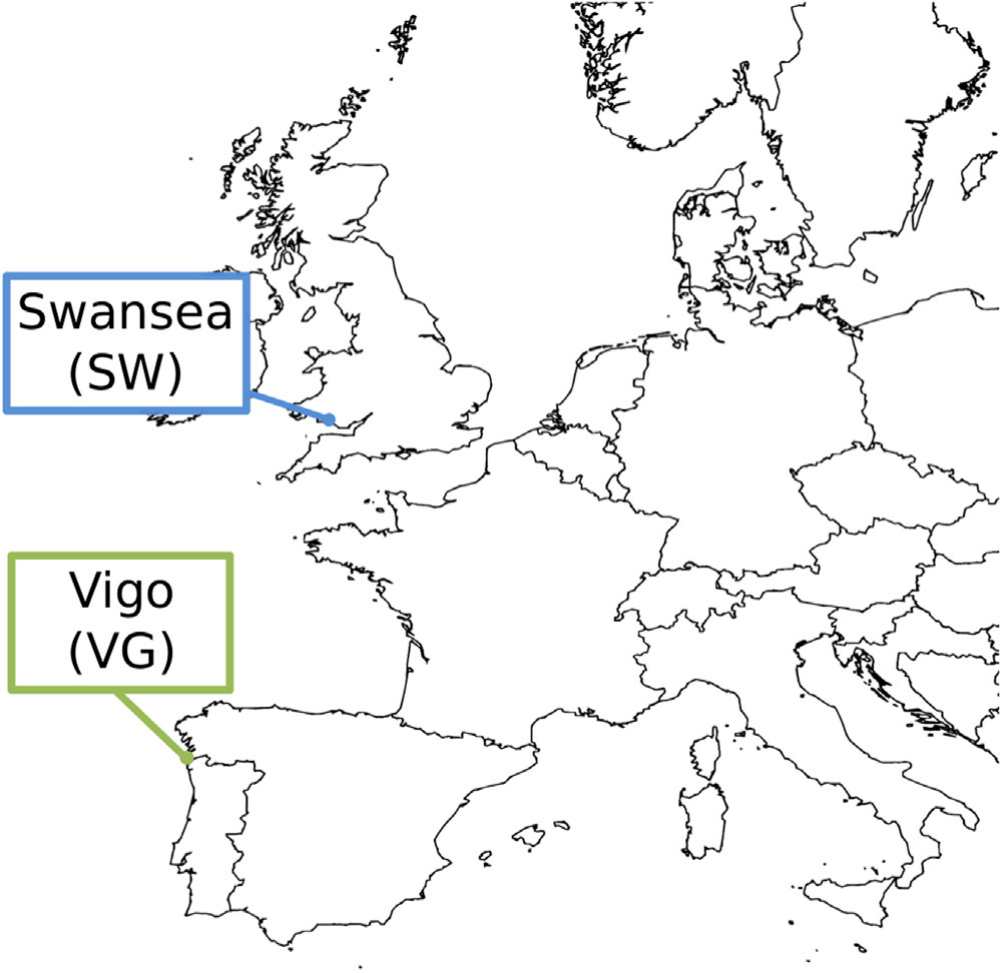
Geographical location of the two sampling areas, Swansea (United Kingdom) and Ría de Vigo (Spain), corresponding to presence of *Mytilus edulis* and *M. galloprovincialis* mussels respectively.

Mussels from *M. edulis* and *M. galloprovincialis* species were collected (end of January 2012) from rocky shores in Swansea (South Wales, UK; lat. 51.567764°, long. −3.976045°) and Ría de Vigo (North-West Spain; lat. 42.104604°, long. −8.898815°) at the end of January of 2012 (Fig. 1).

Mussels were transported to CIM-UVIGO (the marine station of the University of Vigo), and kept in polyurethane boxes (50 L) under the same laboratory conditions for at least 2 months. This was an environment with filtered (20 µm sieve) seawater at a constant temperature of 13 °C (pH = 8.1*,* salinity = 34‰), with water being renewed at a rate of 50 L/h. Added food consisted of a microalgae mix (40% *Isochrysis galbana,* 10% *Tetraselmis suecica,* 20% *Chaetoceros gracilis,* 20% *Phaeodactylum tricornutum* and 10% *Rhodomonas lens*) at 5% tissue dry weight of the mussels excluding shells per day. After 2 months of acclimation, individually collected mussels were processed to obtain two pieces of gonad tissues per mussel. One piece of gonad tissue was immediately snap frozen, labelled and preserved long-term in liquid nitrogen prior to further RNA-seq analysis. A second piece was used to carry out a histological test (based on standard hematoxylin-eosin stain) to assess the sex and stage of gonad maturity as explained in Romero et al. [1]. This procedure was repeated for mussels from both species until samples from 6 individual mussels from each *Mytilus* species were obtained corresponding to mature male individuals for further RNA-seq analysis.

### 2.2 RNA extraction

**Fig. 2:**
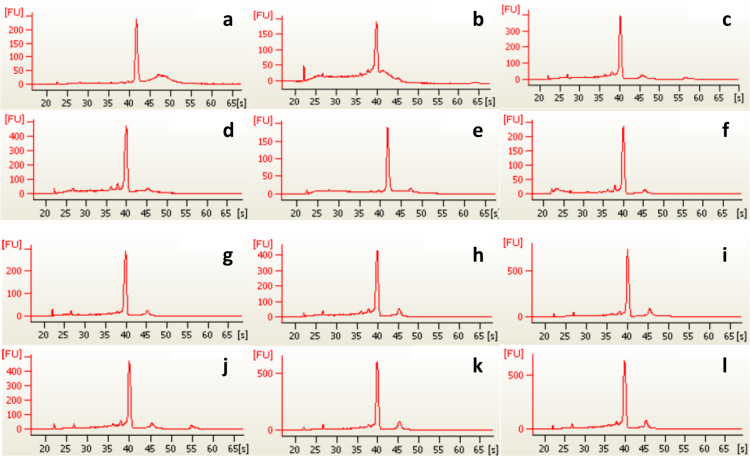
Bioanalyzer profiles for each of the 12 samples of RNA finally selected for further RNA-seq analysis. The first six panels (a)–(f) correspond to results from *Mytilus edulis* individual samples, while the other six below (g)–(l) to *M. galloprovincialis* individual samples. Typical eukaryotic profiles with two peaks of ribosomal RNA were not observed, in common with some other protostomes [2]. However the observation here of similar profiles for all individuals, and a single strong 18S peak without smearing, confirms that good quality RNA has been extracted.

A protocol based on the Qiagen RNeasy® Mini kit (Qiagen, Valencia, CA, USA) with tissue homogenization in QIAshredder columns (Qiagen) was used for RNA extraction. Each sample was treated with DNase and diluted in 35 µl of RNase-free water. The quantification of RNA samples was carried out using a NanoDrop 1000 Spectrophotometer (Thermo scientific, DE, USA), while the RNA quality was assessed in an Agilent 2100 bioanalyzer (Agilent Technologies, CA, USA). Bioanalyzer profiles of the 12 samples are displayed in Fig. 2. The samples were used to make two pools of 6 individuals each, one pool for each of the two *Mytilus* species. 700 ng of RNA per individual sample was used, so each pool contained 4.2 µg of total RNA.

### 2.3 Preparation of mRNA libraries

These were generated using the Illumina Truseq Small RNA Preparation kit (Illumina, CA, USA) according to Illumina׳s TruSeq Small RNA Sample Preparation Guide v2 (low sample protocol). The main steps were (please see more information in the Illumina Support Center https://support.illumina.com):

a) mRNA purification from 4.2 µg of total RNA per pooled sample (see above) using magnetic beads with oligo (dT)
b) fragmentation of purified mRNA by heat incubation
c) synthesis of the first cDNA strand, using random primers and SuperScript II reverse transcriptase enzyme (Invitrogen, CA, USA)
d) second strand synthesis of cDNA
e) generation of blunt-ends
f) adenylation of 3′ ends
g) ligation of specific adapters from Illumina platform and paired-end protocol
h) library amplification by 15 cycles of PCR
i) agarose gel-based selection of libraries with fragments close to 500 bp
j) quality assessment of libraries through Bioanalyzer profiles using a high sensitivity DNA chip. In the two samples (one for *M. galloprovincialis* and another one for *M. edulis*) the size of libraries fits well to the expected size.
k) library quantification by using quantitative PCR with specific primers complementary to the library adapters and KAPA SYBR FAST Universal qPCR Kit (Kapa Biosystems, MA, USA).

Libraries were diluted to 12 pM before sequencing.

### 2.4 Sequencing (Illumina HiScanSQ)

**Fig. 3:**
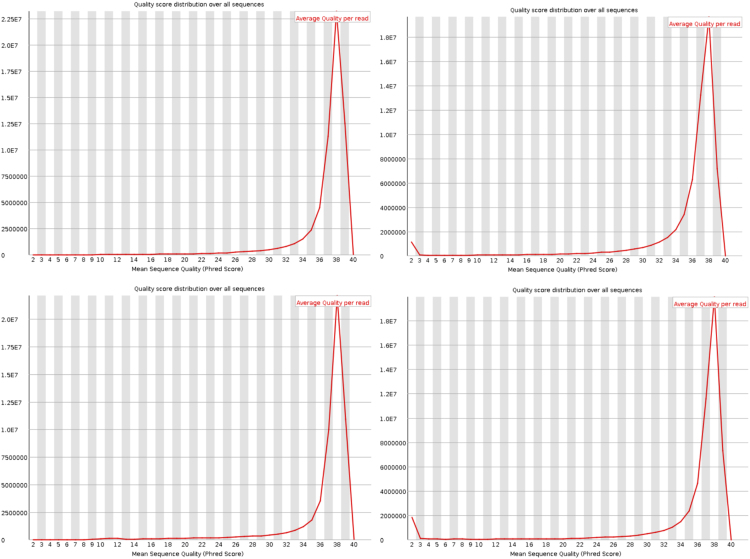
Diagrams showing the quality of reads from each pair of reads (because of the paired-end sequencing approach followed in this experiment) per sample. Top and bottom panels correspond to reads from *Mytilus edulis* and *Mytilus galloprovincialis* samples, respectively. *X*-axis, the mean Phred quality score for each read of 100 bases long (the higher this value, the better the read quality). *Y*-axis, the number of reads.

Each library (one per pooled sample/*Mytilus* species) was analysed in a full line of an Illumina HiScanSQ instrument (Illumina) and using TruSeq SBS v3 chemistry (Illumina) yielding 2 × 100 bases long paired-end reads. After sequencing of cDNA clusters, data (sequencing images) were acquired and analysed by using the Genome Analyzer Sequencing Control Software (SCS 2.6) and Real Time Analyser (RTA 1.6) software from Illumina. The final output consists of two.fastq files per line, each pair corresponding to the full set of reads for the two analysed *Mytilus* spp. The quality control of these nucleotide sequences (paired-end reads) was carried out by using FastQC software (https://www.bioinformatics.babraham.ac.uk/projects/fastqc). A total of 124,102,082 and 111,865,458 reads were obtained from the *M. edulis* and *M. galloprovincialis* samples respectively. The quality of these sequences was assessed by using the Phred-score that indicates the reliability of base-assignments (base-call) for each short read. A short-read Phred quality score ≥20 units was accepted as a good quality-measure for each read, *i.e.* an expected sequencing error of 1% (or lower). In all cases, more than 94% of the total reads met this criterion as is shown in Fig. 3.

An additional filtering step to improve the quality of the sequenced fragments based on 3′end-read trimming was carried out in order to eliminate those low-quality (Phred score <20) nucleotides by using the ConDeTri v2.0 software (https://github.com/linneas/condetri) [3]. If one of the paired-end read was excluded due to a low quality, the other read (single-read) of good quality remained valid for further analyses. A minimum length of 90 nucleotides was necessary to be valid. After applying these filters, there are paired-end reads but also single reads. For the *M. edulis* and *M. galloprovincialis* samples there are 78.85% (69.8% paired/ 9.05% single) and 80.4% (72.9% paired/ 7.5% single) of total reads, respectively, passed both filtering criteria. In summary, after filtering, 187,829,361 reads (79.6% of the total initially generated fragments) were used for *de novo* assembly and generation of a consensus transcriptome for the two analysed samples (mature male gonad) of *Mytilus* spp.

### 2.5 *De novo* sequencing

*De novo* transcriptome assembly was carried out by using Velvet and Oases software [4], [5]. Velvet defines *k*-mers for assembly. A *k*-mer is a stretch of sequence of length *k*, which is used to perform the assembly, so only a piece of each read of length *k* is used to start the assembly. This makes the assembly more efficient and there is less redundancy. In our analysis six different k-mer lengths were evaluated: 55, 59, 63, 67, 71 and 75. It was observed that *k*-mer=63 provided the best result, *i.e.* a good trade-off between specificity and sensitivity. The preliminary assembly of reads obtained after Velvet analysis was later completed by Oases to generate different isotigs. Finally, it clusters the isotigs into small groups called loci, which could be regarded as the consensus transcriptome of samples under study. These loci are a collection of similar sequences (isotigs) derived from the same gene, which might include different splice variants, alleles and partial assemblies of longer transcripts. A total of 97,425 isotigs that were grouped in 49,713 loci were obtained after the assembly (Files S1 and S2). File S2 represents the consensus transcriptome of mature male gonad tissue from *M. galloprovincialis* and *M. edulis*. The average, maximum and N50 length of isotigs is 706, 13604 and 1071 nucleotides, respectively. Headings of each locus sequence include the following information: the locus identifier, number and chosen transcript (isotig) as a representative of that locus, a confidence value which is a measure of the uniqueness of one isotig at that locus. This value taken by an isotig varies between 0 and 1 and indicates how it relates to other isotigs of the same locus. The closer the value to 1, the more similar to other isotigs of the same locus. The final information in the heading is the representative isotig length in base pairs for each locus. In order to evaluate the redundancy of transcriptome assembly, the CD-HIT program (http://weizhongli-lab.org/cd-hit) was used [6]. It was observed that only 756 loci showed significant similarity to any other transcriptome (loci) sequences. This means that the redundancy level is 1.5%, with a 95% of identity and coverage.

### 2.6 Transcriptome annotation

**Fig. 4:**
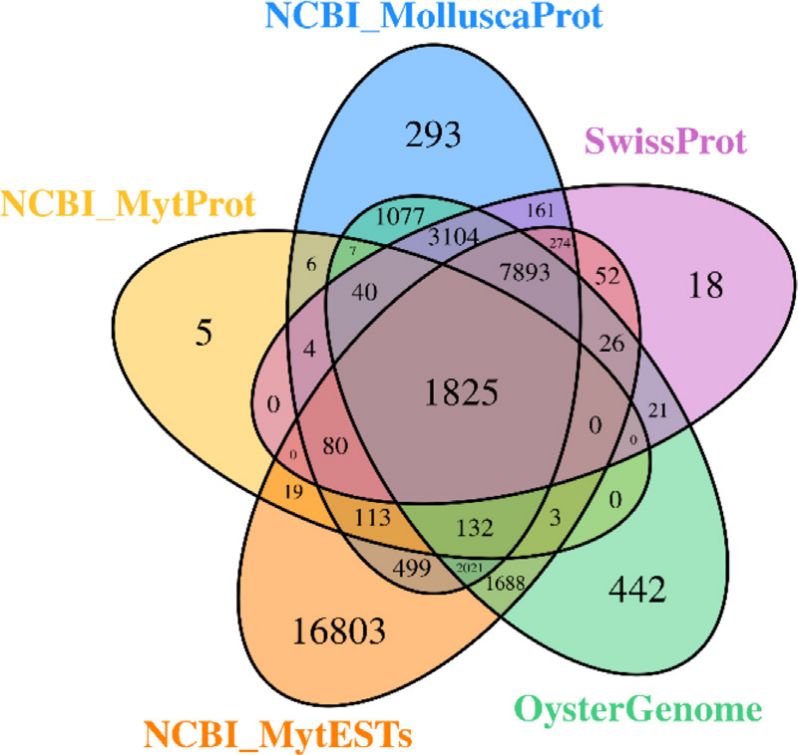
Venn diagram showing the BLAST results of consensus *Mytilus* spp. transcriptome against five databases. The generated consensus transcriptome was annotated against a non-redundant UniProtKB/SwissProt (**SwissProt**) sequence database using the program BlastX and the annotation was repeated against the published genome of another marine bivalve, the Pacific oyster *Crassostrea gigas* (**OysterGenome**), all EST sequences available in NCBI from “Mytilus”[organism] (**NCBI_MytESTs**), and two protein databases with sequences retrieved from NCBI either for “Mytilus”[Organism] (**NCBI_MytProt**) or “Mollusca”Organism (**NCBI_MolluscaProt**) using a threshold *e*-value of 10^−3^. The number of transcripts that have significant hits against the five databases is shown in each intersection of the Venn diagram.

The generated consensus transcriptome (49,713 loci) was annotated against UniProtKB/SwissProt database (number of sequences = 456,613; 2013/11/01) using BlastX. The significance value for alignment was set as 1 × 10^−^^3^. A total of 13,498 transcripts (27.2%) were successfully identified. For comparative purposes, the annotation was repeated against 1) the full published genome of another marine bivalve, the Pacific oyster *Crassostrea gigas* [7], 2) all EST sequences available in NCBI from “Mytilus”[organism] (67,990 sequences; 2014/01/03), by using tBlastX, and 3) two protein databases with sequences retrieved from NCBI either for "Mytilus"[Organism] (6338 sequences; 2014/01/03) or "Mollusca"[Organism] (190,951 sequences; 2014/01/03), using BlastX (see Fig. 4). In all cases an e-value threshold of 1 × 10^−^^3^ was used.

### 2.7 GO annotation and analysis

**Fig. 5:**
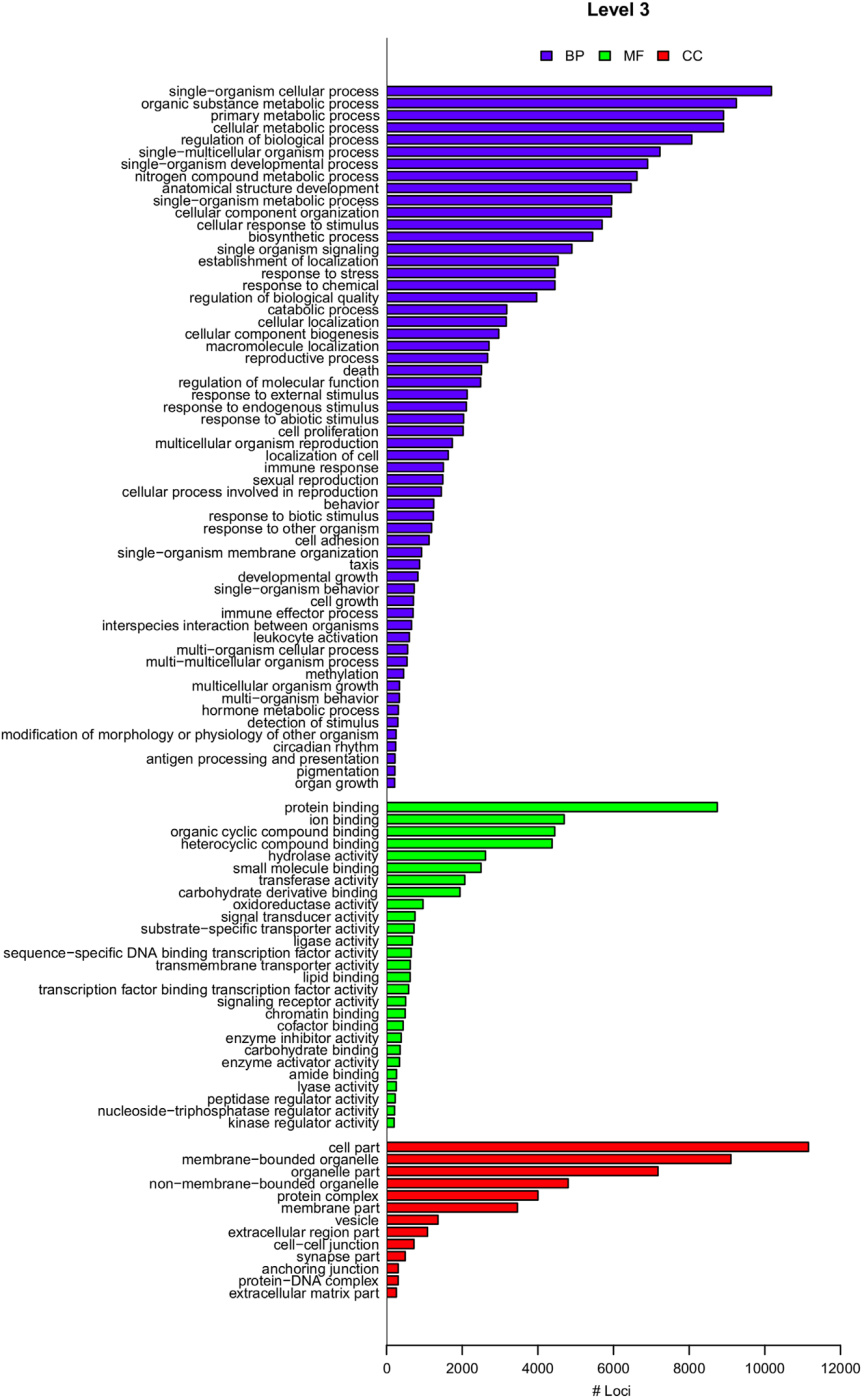
Gene Ontology (GO) term annotations at level 3 of the different ontology categories for the consensus transcripts of *Mytilus* spp. The ontology categories are BP (biological process), MF (molecular function) and CC (cellular component). Only terms annotated in at least 200 loci are shown.

Ontological annotation through Gene Ontology terms was performed using the tool Blast2GO [8] for the consensus *Mytilus* transcriptome (49,713 loci). The starting points were the 13,498 (loci) sequences with significant hits (*e*-value threshold 1 × 10^−^^3^) from BlastX analysis. From these sequences, 13,348 were successfully mapped (*i.e.*, GO terms were extracted from the matching sequences to query resulting from the BlastX analysis), while 12,156 were successfully annotated (File S3). An improvement in the annotation step was reached by running InterProScan (IPSR) 5.0 [9] through Blast2GO. This tool provides functional analysis of proteins by classifying them into families and predicting domains and important sites. With this the number of successful annotations increased to 13,283 loci (File S4). Combined graphs for the full annotated transcriptome were also generated for the most informative levels (level 2 and 3 in tree hierarchy) accounting for GO-term distributions to molecular function (MF), biological process (BP), and cellular component (CC) (see Fig. 5, and also Fig. 2a in Ref. [1]). An enrichment analysis (Fisher׳s exact test) was carried out for those loci that showed significant differences (at least in one isotig within locus) between samples of the two *Mytilus* spp. (see next section). This procedure checked whether Gene Ontology terms are enriched in a test group (those transcripts with significant differential expression in our analysis) when compared to a reference group (the full annotated transcriptome) using the Fisher׳s exact test with a FDR=5%. This analysis provides a list of enriched GO terms (over or under-represented compared to the full transcriptome) associated with those significant transcripts (see Fig. 2b in Ref. [1]).

### 2.8 Differential expression analysis between *Mytilus edulis* and *M. galloprovincialis*

In the present study, there are no biological replicates within each *Mytilus* spp. sample, but rather we followed a pooling approach (RNA was pooled from six individuals for each *Mytilus* spp.). The differential gene expression analysis was carried out by using RSEM [10] combined with EBSeq [11] software. RSEM is first used to quantify expression, then EBseq carries out the differential expression analysis. Statistical methods based on the Negative Binomial (NB) distribution (see [12]) were used to make inferences about differential expression between the *Mytilus* species. A total of 27,233 isotigs (28%; FDR 5%), which corresponds to 14,737 (29.6%; FDR 5%) loci, were found differentially expressed between pooled samples of the two *Mytilus* spp. (*p*<0.05, FDR=5%). If the *p*-value and FDR threshold are set up to 1%, a total of 20,997 isotigs (21.6%) are differentially expressed, which corresponds to 11,335 (22.8%) loci. File S5 reports the expression values for all isotigs (isotigs with zero counts in both conditions were excluded for further analysis) in the two *Mytilus* spp. File S6 reports the calculated statistics for all transcripts (isotigs). For each transcript, four statistics (estimated by EBSeq) are reported: "PPEE", "PPDE", "PostFC" and "RealFC". "PPEE" and “PPDE” are the posterior probabilities that a transcript is equally or differentially expressed between *Mytilus* spp. samples, respectively. "PostFC" and "RealFC" are the posterior and real fold change (*M. edulis* over *M. galloprovincialis* sample) for a transcript. Further details can be found in the readme file, available in the RSEM webpage (http://www.webcitation.org/query.php?url=http://deweylab.biostat.wisc.edu/rsem&refdoi=10.1186/1471-2105-12-323), and EBseq tutorial (http://www.bioconductor.org/packages/devel/bioc/vignettes/EBSeq/inst/doc/EBSeq_Vignette.pdf). File S7 provides the annotation (using BlastX, see details in the section above “transcriptome annotation”) for all transcripts (loci) where a significant result was found, and annotation was feasible (e-value threshold 1 × 10^−^^3^).
